# Technical aspects of reconstruction for inadequate left atrial cuff in lung transplantation

**DOI:** 10.1186/s13019-023-02451-7

**Published:** 2023-12-08

**Authors:** Sevinc Citak, Murat Ersin Cardak

**Affiliations:** Department of Thoracic Surgery, Kartal Kosuyolu High Specialization Education & Research Hospital, Istanbul, Turkey

**Keywords:** Reconstruction, Inadequate left atrial cuff, Lung transplantation

## Abstract

**Objective:**

Lung transplantation is the only life-saving treatment for lung diseases that do not respond to medical treatment. Heart-lung harvesting requires a careful procedure to protect an adequate donor left atrial cuff around the junction of the superior and inferior pulmonary veins. This study aims to describe inadequate left atrial cuff during harvest and techniques of reconstruction at the threshold of literature.

**Methods:**

Left atrial cuff complications were retrospectively analyzed in consecutive lung transplant procedures between December 2016 and December 2021. Donor and patient demographics, reconstruction material and method of application and postoperative follow-up were examined.

**Results:**

In the study period, 84 consecutive lung transplant procedures were performed. Reconstruction of the inadequate left atrial cuff was 3.7% (6/162) for atrial anastomoses. However, the inadequate left atrial cuff was 9.1% (5/55) in heart-lung harvesting. Donor aorta graft was used in 4 patients and Dacron mesh was used on the bilateral atrial cuff in one patient. Hospital mortality occurred in one patient. One patient died 6 months later due to antibody-mediated rejection. The follow-ups of the other three patients are continuing without any problems.

**Conclusions:**

Inadequate left atrial cuff complications occurring in heart-lung harvest seem to be more common than in the literature. Techniques of reconstruction for the inadequate left atrial cuff is vital for the patient who has reached irreversible progress in surgery for the recipient, as well as increasing the number of organs.

## Introduction

Lung transplantation is the only life-saving treatment for patients with end-stage lung diseases. A successful transplant begins days and months before the transplant with the pretransplant assessment day and then proceeds with suitable donor and recipient matching [1]. The number of patients waiting for lung transplantation has been increasing over the years, and the gap between the number of patients waiting for transplantation and the availability of lung donors is gradually increasing. The importance of harvesting heartbeating brain dead donors has become increasingly important, as there is no or limited living donor transplantation for organs such as the lungs and heart, where there is insufficient donation.

The donor is evaluated in many parameters and matched with the appropriate recipient, such as age, sex, history, laboratory results, cause of death and radiological imaging. After matching the appropriate donor-recipient, the harvesting process is started [2]. Thoracic harvest, a surgical procedure that begins after the final decision to use organs, is made on the operating table of the heart and lung teams and ends in a short time.

Harvest is a keystone even if it takes a short time during the transplant process. Various complications can occur that can lead to inability to use the organ or organ rejection even if it is used due to anatomical variations or deficiencies in surgical technique. Particularly in donors in which the heart and lung will be used, there may be difficulties in providing enough tissue for the two organs in the sharing of left atrial tissue [3].

The lack of surgical practice can be considered one of the important causes that can reduce the success of successful heart-lung harvesting, particularly in centers with low-volume heart and lung transplants. Separate harvesting of the heart and the lungs from one donor for two recipients is complicated due to reaching a consensus on the left atrial cuff. Techniques for coping with developing complications are limited in the literature. This study aims to describe our experience against surgical technical complications during harvest at the threshold of literature.

## Methods

Between December 2016 and December 2021, a total of 5 patients with inadequate left atrial cuff in 84 consecutive lung transplant procedures were analyzed retrospectively. The study protocol was approved by Local Ethics Committee (No: 2022/11/614). The study was conducted in accordance with the principles of the Declaration of Helsinki.

### Donor selection

Matching between donor lungs and the recipient was based on blood group compatibility and predicted total lung capacity (pTLC) by height and age. The criteria for donor selection included PaO_2_/FiO_2_ value, smoking history, donor age, chest X-ray, tomography, cultures, bronchoscopy findings and sputum characteristics. We described donor management and the selection process in our previous article [4].

### Harvesting technique

Lung procurement and preservation followed standard procedures [5]. Harvest is performed by the harvest team of the transplant center in our country. When the harvest team goes to the donor center, they first perform bronchoscopy. If bronchoscopy is appropriate, the operation phase is started. Lung examination begins with standard procedures. Atelectasis-hematomas are detected and performed in the recruitment maneuver. To evaluate elasticity, the collapse test is performed with the help of anesthesia.

A purse-string suture is placed in ascending aorta and the inserted cardioplegia cannula is fixed with the prepared suture. We insert a cannula into the main pulmonary artery just after the pulmonary valve, allowing drainage of the right and left lungs. The reason why we place it close to the pulmonary valve is that our heart team primarily repairs the pulmonary artery after the cannula is removed so that it does not stay in the anastomosis line of the heart team and sufficient tissue is provided in the heart transplant, avoiding unnecessary shaving. Then, an incision is made with a sharp scalpel in the middle of the purulent sutures in the pulmonary artery, the incision is widened, the cannula is inserted, fixed and then washing is started. While the patient is connected to the ventilator, washing at 5 cm H_2_O positive end-expiratory pressure ensures that the washing solutions are spread more homogeneously and there is no ponding.

The aortic cross-clamping is then placed and both cardioplegia and pulmoplegia lines are opened. Harvesting is started following the completion of lung and heart washings and cooling. First, the inferior vena cava is dissected up to the right inferior pulmonary vein. The heart is exposed by raising the apex to the cephalad position. After the apex is raised to the cephalad position, the incision made from the middle of the left atrium to the left pulmonary vein is widened with a scalpel by paralleling it to the left pulmonary vein.

The inside of the left atrium is best seen by the surgeon on the donor’s left. The left atrial incision is extended to the intra-atrial groove (Waterson). We do this widening cut from the outside. A distance of at least 1 cm should be left for the heart and lungs in the left atrium with an adequate rim. It is very important not to stretch the heart too much during cutting because if it is cut by pulling too much, there will not be enough lung anastomosis tissue when it is released. Scissors should always be held horizontally. During this time, care should be taken not to cut the pulmonary artery.

The heart should be placed in its neutral position and cut to leave enough aorta and superior vena cava tissue for the heart and the two vessels should be carefully separated from the right pulmonary artery. The pulmonary artery is then divided and ascending aorta divided. Pericardial attachments are dissected and the heart, freed from all connections, is placed on the back table and the heart harvest is completed. The pulmonary artery is then divided. After heart harvest, the bilateral lung is removed unblocked. Then, the lung is placed in an organ protection bag.

### Repair and reconstruction

**Fig. 1:**
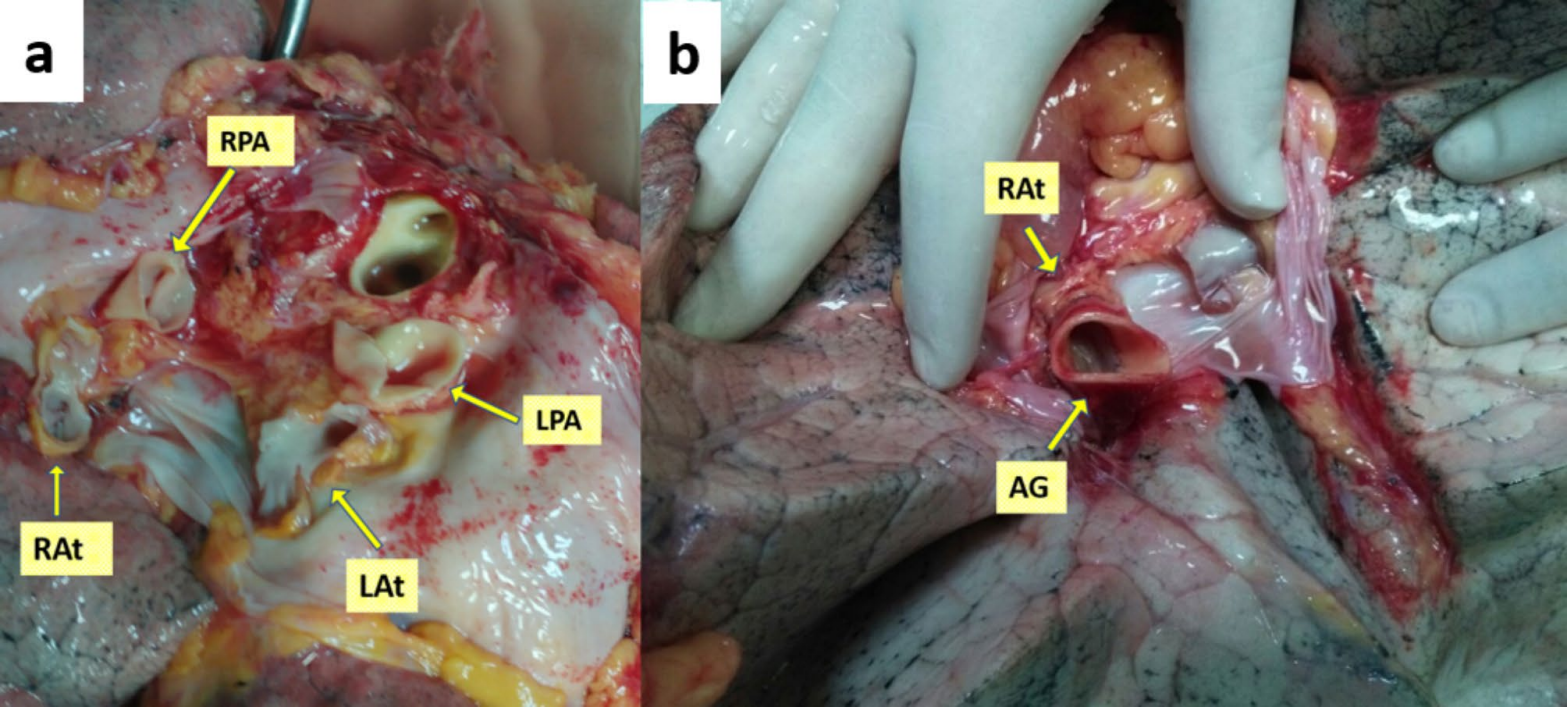
Intraoperative photographs showing inadequate left atrial cuff and aortic graft reconstruction. **(a)** Insufficient left atrial cuff; RPA: right pulmonary artery, LPA: left pulmonary artery, RAt: right atrium, LAt: left atrium. **(b)** Left atrium repaired with aortic graft, AG: aortic graft, RAt: right atrium

In the presence of an inadequate atrial cuff in its anterior and posterior wall, it was augmented with a donor aortic graft. The entire thoracic aorta was routinely harvested from the donor and transferred with the lung in an organ preservation solution for spare graft tissue that we could use for injuries that might occur during harvest or on the back table. The donor aorta was shaped to complete the deficiency from an undamaged and smooth surface with no outflow of spinal arteries after the aortic arch. On the back table, aortic graft and donor pulmonary vein anastomosis were performed using a 5 − 0 polypropylene running suture to form a separate atrial cuff. The new aortic-cuffed atrium was sutured to the recipient’s atrium using a 4 − 0 polypropylene running suture (Fig. 1).

**Fig. 2:**
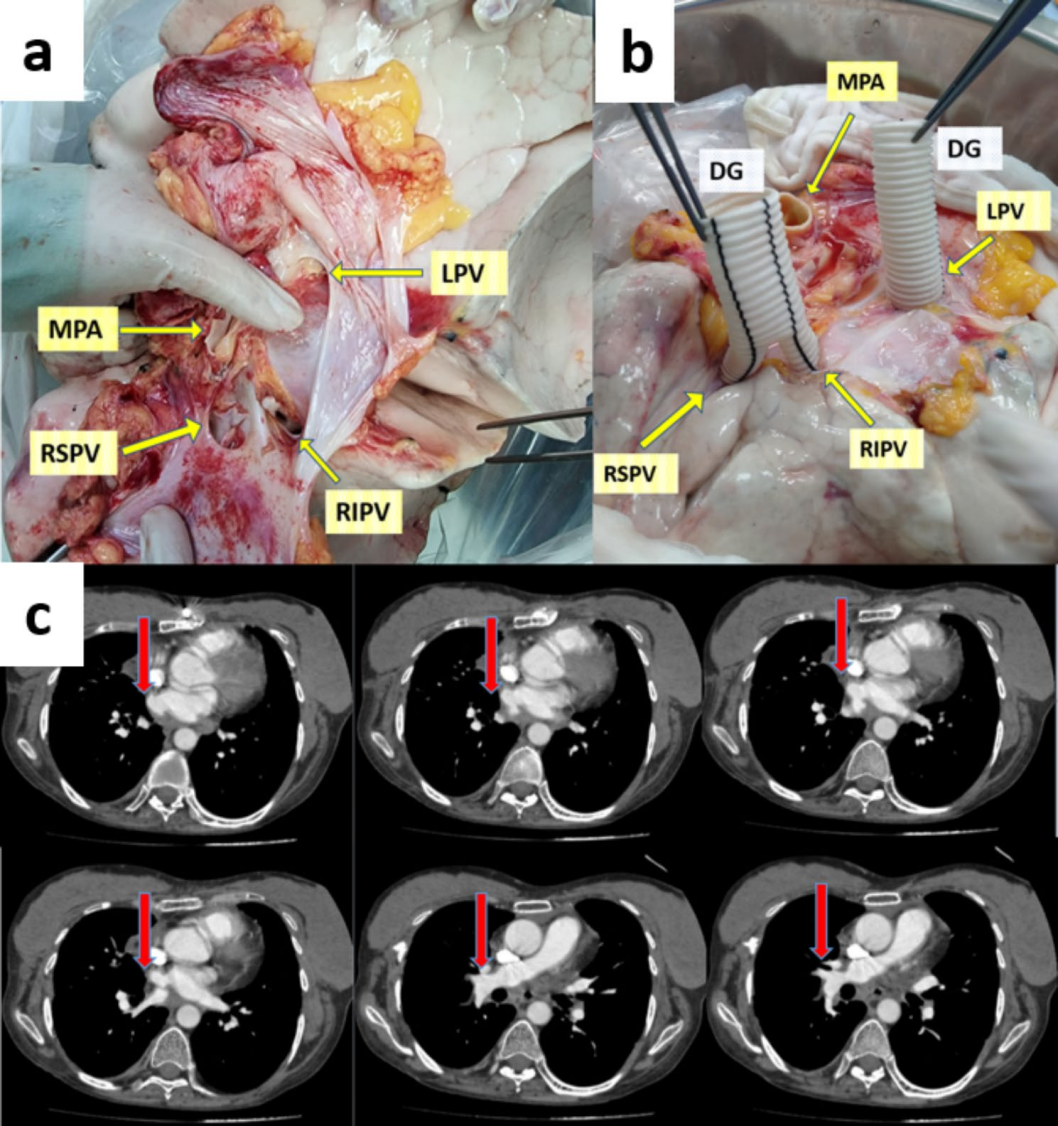
Inadequate cuff, intraoperative photographs showing reconstruction with dacron mash, and postoperative Pulmonary Computed Tomography Angiography images. (a) Insufficient left atrial cuff: RIPV: Right inferior pulmonary vein, LPV: Left pulmonary vein, MPA: main pulmonary artery, RSPV: right superior pulmonary vein. (b) Left atrium repaired with Dacron Mash, RIPV: right inferior pulmonary vein, PV: left pulmonary vein, MPA: main pulmonary artery, RSPV: right superior pulmonary vein, DG: dacron graft. (c) Anastomosis line (arrow) in the third month postoperative Pulmonary Computed Tomography Angiography

A bifurcated Y-shaped Dacron conduit (Hemagard knitted vascular graft; Maquet) patch was used to separate the superior and inferior pulmonary veins. The anastomosis was performed on the back table by cutting the Dacron graft limb to the appropriate length to obtain the additional length of both the inferior and superior pulmonary veins. The reconstructions were performed using a 5 − 0 polypropylene running suture. Anastomosis between the Dacron graft common body and the recipient’s atrium was performed using a 4 − 0 polypropylene running suture. During the completion of the anastomoses, retrograde and antegrade air evacuation was performed. All anastomoses were then carefully examined to detect any tension, twisting, narrowing, or bleeding. Transesophageal echocardiography was performed perioperatively for anastomosis control and control was obtained with pulmonary CT angiography in the postoperative period (Fig. 2).

Anticoagulant therapy was not given in the patient we used for the aortic graft and acetylsalicylic acid was used in the patient we used for the Dacron mesh.

## Results

In the study period, 162 atrial anastomoses were performed in 84 consecutive lung transplant procedures. Reconstruction was required in 3.7% (6/162) of these anastomoses. Atrial reconstruction was performed in 5 (5.9%) of 84 patients. However, the inadequate atrial cuff was 9.1% (5/55) in heart-lung harvesting. Inadequate atrial cuffs were on the left side of the anastomosis in all cases, but they were bilateral in one patient. The types of reconstruction of the left atrial cuff were as follows: four patients were reconstructed by the aorta and one by a Dacron mesh on the bilateral veins. All donors requiring atrial cuff reconstruction underwent heart extraction. None of the donor lungs were discarded because of problems with the adequacy of the left atrial cuff.

Donor and recipient demographics and atrium reconstruction information are given in Table 1. There were no unused donor lungs due to inadequate left atrial cuff. Of the recipients, two were female and three were male. In terms of recipient-donor gender, four were matched, while a female donor lung was used for a male recipient. The mean ischemia time was 327 (210–406) minutes for the first lung and 475 (360–560) minutes for the second lung. The extracorporeal membrane oxygenation (ECMO) use rate was 60% (3 in 5). Heart harvesting was present in all cases requiring reconstruction. Grade 3 primary graft dysfunction developed in three out of five patients.

**Table 1:** Donor and recipient demographics

	Patient 1	Patient 2	Patient 3	Patient 4	Patient 5
**Donor**					
**Age**	47	43	54	32	41
**Sex: recipient/donor**	F/F	F/F	M/M	M/M	M/F
**Height, (** ***cm*** **)**	165	160	185	175	165
**BMI**	27,5	25,3	23,3	27,7	22
**Cause of death**	CVD	CVD	CVD	CVD	CVD
**Donor intubation, day**	4	7	4	1	2
**Preprocurement PaO**_**2**_ **/FiO**_**2**_	320	390	316	296	414
**Heart harvesting**	Yes	Yes	Yes	Yes	Yes
**Recipient**					
**Age (** ***years*** **)**	55	33	61	55	36
**Sex**	F	F	M	M	M
**Underlying disease**	Sarcoidosis	Bronchiectasis	COPD	IPF	Silicosis
**Echocardiography**					
**RV dilatation**	-	-	+	-	+
**TAPSE, mm**	1,3	1,9	1,8	2,5	2,4
**RHC**					
**PAPs, mmHg**	29	54	80	40	40
**PAPm, mmHg**	18	35	51	29	23

**Abbreviations:** F: female; M: male; BMİ: body mass index; CVD: Cerebrovascular disease; PaO2/FiO2: partial pressure of oxygen in arterial blood / fraction of inspiratory oxygen concentration; COPD: chronic obstructive pulmonary disease; IPF: idiopathic pulmonary fibrosis; RV: right ventricle; TAPSE: tricuspid annular plane systolic excursion; mm: millimeter; RHC: right heart catheterization; PAPs: pulmonary antery pressure systolic; PAPm: pulmonary antery pressure mean.

In the analysis of the amount of use of blood products, the use of erythrocyte suspension in the first 24 h in three patients who used ECMO was 10/11/15 units, respectively. In the two patients who did not use ECMO, the number of erythrocyte suspensions used in the first 24 h was 0/3 units. We observed that the use of erythrocyte suspension was significantly higher in the group using ECMO and the use of fresh frozen plasma and platelet suspension was significantly increased in the group using ECMO compared to the group not using ECMO (Table 2).

**Table 2:** Intraoperative data and outcomes

	Patient 1	Patient 2	Patient 3	Patient 4	Patient 5
**Lung transplantation type**	Bilateral	Bilateral	Bilateral	Bilateral	Bilateral
**ECMO use**	+	+	+	-	**-**
**Severe PGD (grade 3)**	+	+	-	-	+
**Number of RBC transfusions on perioperative/ in the postoperatively first 24 h**	10/0	4/7	15/0	0/0	1/2
**Number of platelet transfusions on perioperative/ in the postoperatively first 24 h**	2/0	0/5	0/4	2/0	0/4
**Number of plasma transfusions on perioperative/ in the postoperatively first 24 h**	3/4	2/8	10/6	3/0	5/3
**Reconstruction side**	L	L + R	L	L	L
**Reconstruction type**	Aortic graft	Dacron graft	Aortic graft	Aortic graft	Aortic graft
**Graft ischemic time**	R;390 L:540	R: 406 L: 525	R: 390 L: 560	L: 210 R: 390	L: 240 R: 360
**Rethoracotomy**	-	+	-	-	-
**ICU length of stay (** ***days*** **)**	12	18	4	3	9
**Hospital length of stay (** ***days*** **)**	12	53	19	24	45
**Survival (** ***month*** **)**	1	6	36	61	43
**Cause of death**	Acute rejection	AMR	Alive	Alive	Alive

**Abbreviations:** ECMO: Extracorporeal membrane oxygenation; L: left; R: right; PGD: Primary graft dysfunction; RBC: red blood cell; ICU: intensive care unit; AMR: antibody-mediated rejection

Hospital mortality occurred in one patient. One patient died six months later due to antibody-mediated rejection. The follow-ups of our other three patients are ongoing without any problems. The patient with in-hospital mortality was diagnosed with sarcoidosis and underwent bilateral lung transplantation without the use of ECMO. The perioperative period was normal except for an insufficient atrial cuff. While the patient was taken from the operating room to the intensive care unit, hyperacute rejection developed and pulmonary edema fluid came from the endotracheal tube. Therefore, the patient underwent venovenous ECMO. The patient died on the 12th day.

The patient who died at the 6th month was the patient who used bilateral Dacron grafts (Fig. 2). Postoperative (Po) revision was performed in the early period due to hemorrhage. The patient with a diagnosis of bronchiectasis had severe pleural adhesions due to frequent infections. The patient who was transplanted under venoarterial ECMO had intense bleeding foci. Due to hemodynamic instability, the patient was taken to the intensive care unit with central ECMO delayed chest. The patient’s ECMO was weaned after being followed up in the intensive care unit for one day and stabilized. Bleeding diathesis was regulated. The patient was taken for revision on the 3rd day of the Po. The patient was taken to the service on Po day 6 and discharged on day 49. She was admitted to the ward with sudden onset of shortness of breath while her clinic was stable in the 6th month after the transplant. The diagnosis of antibody-mediated rejection was made by bronchoscopic biopsy. Plasmapheresis and IVIG treatment were initiated and the patient died due to failure to respond to the treatment. The follow-up of our other three patients continues without any problems.

## Discussion

In our study, the incidence of inadequate atrial cuff was 9.1% in heart-lung harvesting. There are a few cases reports about the reconstruction of an inadequate atrial cuff. Therefore, the rate associated with the incidence of atrial cuff reconstruction in lung transplantation is unknown. Takahiro et al. reported that the reconstruction of the insufficient atrial cuff rate was 4.1% (17 patients) in 405 cases of lung transplantation [3]. According to this publication, our rate was high. We believe that the reason for this high rate is that Turkey is in the category of developing countries for lung transplantation. The inadequate atrial cuff was not observed in patients who had lung harvesting without heart harvesting. Lung harvesting is a complex surgical procedure. This process becomes more complex when heart harvest is added to lung harvest. When the heart is extracted only, the pulmonary veins are transected. If the lung is used for a separate recipient, the sufficient rim of the left minimum 1 cm atrium should be left as a cuff for the lungs. Surgeons who perform lung and heart harvesting for the first time involuntarily tend to the pulmonary vein. To avoid complications, it is important that the teams communicate with each other before the harvest and go over the procedure verbally. It is necessary to proceed with millimetric scissor incisions from the inner face of the heart and if necessary, a guide can be created with a thoracic surgeon with forceps.

An “inadequate” donor left atrial cuff is a more common technical consideration in lung transplantation than estimated; therefore, overcoming this problem is crucial for salvaging. The pericardial patch is mostly preferred in the reconstruction of the insufficient atrial cuff [3].

The donor iliac vein, superior vena cava and pulmonary artery are less frequently used for reconstruction of the insufficient atrial cuff [6–8]. We preferred the aorta, which has much elastic tissue and is not fragile. The surgically less fragile tissue was easily reconstructed, not allowing the blood to swirl inside. The point to be considered in the use of the aorta, which is accustomed to working at high pressure (systemic circulation), can shrink when exposed to low pressure in the atrium (pulmonary circulation). This shrinkage should be taken into account, particularly when the aorta is intended to be used as a circular graft. Another advantage is that we did not need the autologous tissue anticoagulant used in synthetic grafts. The surgically less fragile tissue was easily reconstructed, not allowing the blood to swirl inside. The point to be considered in the use of the aorta, which is accustomed to working at high pressure (systemic circulation), can shrink when exposed to low pressure in the atrium (pulmonary circulation). This shrinkage should be taken into account, particularly when the aorta is intended to be used as a circular graft. In cases where the upper and lower pulmonary veins were completely separate, a bifurcated Y-shaped Dacron conduit was used to reconstruct the anatomical shape.

The use of anticoagulants was a controversial decision after the reconstruction. Superior vena cava reconstructions in which complete resection of the vessel with prosthetic reconstruction is required are insually accomplished with synthetic grafts [hard polytetrafluoroethylene (PTFE)] or a bovine pericardial tube. It allows excellent long-term patency without subsequent anticoagulation [8–12]. Takahiro et al. stated that they did not use anticoagulants in atrium reconstructions using the pericardium [3]. We did not give anticoagulant treatment to our patients in whom we had 4 aortic grafts. In the patient in whom we placed bilateral Dacron grafts, the decision was more difficult because a synthetic graft was used. Necessary anticoagulation was provided with bivacard for the patient already in the central ECMO support and he was monitored with ACT and aPTT. In the early period after ECMO waening, the patient used 81 mg of acetylsalicylic acid (ASA). Because occlusion was not detected in the controls who underwent pulmonary CT angiography, ASA treatment was stopped in the early period. Postoperative follow-ups were performed with pulmonary CT angios. We did not give anticoagulant treatment to our patients in the long-term follow-ups, since there was no insufficiency in the flow. There were no long-term complications related to anastomoses.

## Conclusion

Inadequate left atrial cuff complications occurring in heart-lung harvest seem to be more common than in the literature. There are no problems with teams that make frequent common inferences. To avoid these problems, it is important that the teams communicate with each other before the harvest and go over the procedure verbally. Reconstruction of techniques for the inadequate left atrial cuff is vital for the patient who has reached irreversible progress in surgery for the recipient as well as increasing the number of organs.

## Data Availability

All data generated or analyzed during this study are included in this published article.
